# Sexual experiences, attitudes, enjoyment and regret in the parent and offspring generations of the Avon Longitudinal Study of Parents and Childhood (ALSPAC): 2022 data sweep.

**DOI:** 10.12688/wellcomeopenres.23059.1

**Published:** 2024-11-12

**Authors:** Yasmin Iles-Caven, Jean Golding, Carol Joinson, Abigail Fraser, Kate Northstone

**Affiliations:** 1 Population Health Sciences, Canynge Hall, 39 Whatley Rd, University of Bristol Medical School, Bristol, England, BS8 2PS, UK; 2 Population Health Sciences, Oakfield House, Oakfield Grove, University of Bristol Medical School, Bristol, England, BS8 2BN, UK

**Keywords:** ALSPAC, sexual debut, sexual attitudes, sexual history, sexual enjoyment, sexual regret, sexual experiences

## Abstract

The aim of this data note is to describe data collected in 2022 on sexual history, attitudes, enjoyment and regret. Data were collected from mothers (age range 47–75 years (mean 60.0), n = 4653) their partners (age range 47-83 years (mean 62.9), n= 1945) and offspring (aged ~30 years, females n= 2702, males n=1366) in the Avon Longitudinal Study of Parents & Children (ALSPAC).

Many of the questions asked are identical, or similar, to those collected in the British NATSAL (National Surveys of Sexual Attitudes & Lifestyles) surveys. Repeating the same questions in both ALSPAC generations allows for direct inter-generational comparisons within ALSPAC as well as across studies. Areas covered include age at sexual debut; having drunk alcohol, used drugs or contraception at sexual debut; the circumstances under which participants met their first sexual partner; sexual orientation; the Brief Sexual Attitudes Scale; regret at first sexual experience, lifetime experiences of sexual regret and the degree of regret, as well as the reason(s) for that regret; number of sexual partners both in the last two years and over their lifetime; current frequency and enjoyment of sex.

ALSPAC provides a rich resource of data collected on a wide variety of topics including details of the participants’ environment, lifestyle, physical and mental health over the life span, including sexual experiences collected retrospectively from the parents, and from the age of 11 in the offspring. There are thus many opportunities for research on a wide variety of topics related to risky sexual behaviours, sexual health, functioning and well-being.

## Background

Early adolescent sexual activity is often considered to be a risky behaviour because it may result in sexually transmitted infections (STIs) or teen pregnancy. In addition, there is evidence that early sexual debut has detrimental effects on the development of self-concept, self-esteem, feelings of regret, and on mental health, relationship skills and sexual functioning in later life (e.g. Kahn & Halpern, 2018; Mårdh *et al.*, 2000; Meier, 2007; Sandfort *et al.*, 2008). Early sexual debut is also associated with previous sexual abuse and with later risky behaviours such as higher numbers of sexual partners over the life course, smoking, drug and alcohol use and antisocial behaviour (e.g. Kastbom *et al.*, 2015). Research has mostly focussed on adolescents and young adults (the latter often of college students), but there is increasing interest in the past and current sexual attitudes, behaviours and activities of middle aged and older people (e.g. Hinchliff *et al.*, 2021).

The Avon Longitudinal Study of Parents & Children (ALSPAC) has collected data on sexual experiences and behaviours in both the parent and offspring cohorts longitudinally over the last 30-years. These data are described in detail for the mothers and their partners ( Iles-Caven & Golding, 2024a) and for the offspring from the age of 11 ( Iles-Caven & Golding, 2024b). This data note describes the data subsequently collected from both generations in 2022, focussing on questions concerning sexual attitudes, behaviours, enjoyment and regret. It explores the differences in reporting between the two generations and between the sexes (for the offspring that is sex assigned at birth).

## Methods

### Participants

***The Avon Longitudinal Study of Parents and Children (ALSPAC)*.** All pregnant women (G0 cohort) resident in the Avon area of south-west England with expected dates of delivery between April 1991 and December 1992 inclusive were invited to enrol in ALSPAC. The initial number of pregnancies enrolled was 14,541, resulting in 13,988 infants who were alive at 12 months of age. Additional phases of enrolment from the age of 7 years are described in more detail in the cohort profile papers ( Boyd *et al.*, 2013; Fraser *et al.*, 2013; Major-Smith *et al.*, 2023; Northstone *et al.*, 2019; Northstone *et al.*, 2023). The total sample size for analyses using any data collected after the age of seven is 15,447 pregnancies, resulting in 15,658 fetuses. Of these, 14,901 children (G1 cohort) were alive at 1 year of age. Some women enrolled with more than one pregnancy, consequently the number of unique mothers who were enrolled was 14,833 in total. G0 partners were invited to complete questionnaires by the mothers at the start of the study but they were not formally enrolled at that time. Note that ‘partners’ were usually the biological father (at least around initial enrolment), but subsequently may be a stepfather, father-figure, or same-sex partner (confirmed change of partner has been noted in only 4.4%). 12,113 G0 partners have been in contact with the study by providing data and/or formally enrolling when this commenced in 2010. 3,807 G0 partners are currently enrolled (recruited via the mother or the study child) ( Northstone *et al.*, 2023), and may not necessarily still be with the study mother.

Since 2014, study data are collected and managed using REDCap electronic data capture tools hosted at the University of Bristol. REDCap (Research Electronic Data Capture) is a secure, web-based software platform designed to support data capture for research studies ( Harris *et al.*, 2009). For those participants who prefer to complete paper versions these are also offered. Please note that the study website contains details of all the data that is available through a fully searchable data dictionary and variable search tool: http://www.bristol.ac.uk/alspac/researchers/our-data/.

### Data Collection in 2022

A questionnaire was administered to the G0 parents between June 2022 and March 2023. Response rates for the mothers was 50% of those invited (n=4653) and for the partners 51% of those invited (n=1945).

The G0 questionnaire had eight sections: Section A membership of different clubs and organisations over their lifetime; Section B physical exercise pre- and post-pandemic; Section C social support networks and details of the help they may have received from the people around them; Section D attitudes and beliefs: optimism/pessimism (LOT-R scale; Scheier *et al.*, 1994), altruism (Ruston scale; Rushton *et al*., 1981), gratitude ( McCullough *et al.*, 2002), forgiveness ( Berry *et al.*, 2005), self-efficacy ( Romppel *et al.*, 2013), and meaning in life ( Steger *et al.*, 2006); Section E internal home environment including cooking methods, types of heating, ventilation, the presence of mould/damp, pests and pets; Section F sexual attitudes and experiences; Section G smoking, cannabis and other drugs and alcohol intake (as a teenager, in their early 20s, pre- and post-pandemic) and Section H reproductive history.

The initial invitation email to complete the Summer 2022 G1 offspring questionnaire was sent in late May 2022. This questionnaire differed from the parent version in that it had 13 sections. In addition to the content included in the parental questionnaires, there were sections on body image and separate sections with more detailed questioning on smoking, e-cigarette use, cannabis, CBD products, other drugs and alcohol. It omitted the section on reproductive history. The response rate was 4068 (45% of those invited).

Identical sets of questions relating to sexual behaviours and experiences (Section F) were asked of both G0 and G1 generations within these questionnaires.

 Section F was entitled “Sexual Attitudes and Experiences”, with the preamble:

“ *The questions in this section are about your attitudes to sex and sexual experiences. We know that this can be quite a sensitive topic and therefore want to reassure you that all your answers are completely confidential. Where we refer to sexual intercourse or 'having sex' please include vaginal, oral or anal sexual intercourse*”.

The wording of the questions and their variable names are listed in Table 1. Here we describe the questions included and the potential responses in the order in which they were presented within the questionnaire.

**Table 1.** The questions (Section F: Sexual attitudes and experiences) administered via self-completion questionnaires to the Parent (G0) and Offspring (G1) ALSPAC cohorts at the 2022 sweep with their variable names. Those prefixed DV = derived variable.

DV = derived variable	G0 Mothers	G0 Partners	G1 Life at 30+
F1. Which of the following best describes your sexual orientation? Gay/Lesbian, Bisexual, Pansexual, Asexual, Heterosexual/Straight, None of these (please describe), Don’t know, Prefer not to answer	MA7010	FPE7010	YPK5510
F1a. On a scale of 0–10, please indicate your attraction to men and women with 0 being ‘only men’, 10 being ‘only women’, and 5 being equally attracted to both. If you can’t or don’t wish to answer this question, please cross one of the other options below: Gender/sex is not important to me; Don’t know; Prefer not to answer.	MA7020	FPE7010	YPK5520
F2. *Listed below are several statements that reflect different attitudes about sex. For each statement, please say how much you agree or disagree with that statement.* *Strongly agree; Moderately agree; Neither agree nor disagree; Moderately disagree; Strongly agree.*			
F2a: Casual sex (e.g. a one-night stand) is acceptable	MA7030	FPE7030	YPK5530
F2b: It is okay to have ongoing sexual relationships with more than one person at a time.	MA7040	FPE7040	YPK5540
DV: sexual attitudes scale- permissiveness score	MA7041	FPE7041	YPK5541
DV: sexual attitudes scale- permissiveness categories	MA7042	FPE7042	YPK5542
DV: sexual attitudes scale- permissiveness, number of missing items	MA7043	FPE7043	YPK5543
F2c: Sex is the closest form of communication between two people	MA7050	FPE7050	YPK5550
F2d: Sex is a very important part of life	MA7060	FPE7060	YPK5560
DV: sexual attitudes scale- communion score	MA7061	FPE7061	YPK5561
DV: sexual attitudes scale- communion categories	MA7062	FPE7062	YPK5562
DV: sexual attitudes scale- communion, number of missing items	MA7063	FPE7063	YPK5563
F2e: The main purpose of sex is to enjoy oneself	MA7070	FPE7070	YPK5570
F2f: Sex is primarily a bodily function, like eating	MA7080	FPE7080	YPK5580
DV: sexual attitudes scale- instrumentality score	MA7081	FPE7081	YPK5581
DV: sexual attitudes scale- instrumentality categories	MA7082	FPE7082	YPK5582
DV: sexual attitudes scale- instrumentality, number of missing items	MA7083	FPE7083	YPK5583
F2g: Sex should be reserved for marriage	MA7090	FPE7090	YPK5590
F2h: A central purpose of sex is to have children	MA7100	FPE7100	YPK5600
F3: How old were you when you **first** had sex? If you can’t remember exactly, please give your best guess. Or tick: Prefer not to answer; I have not had sex	MA7110	FPE7110	YPK5610
*F4: How did you meet the person with whom you first had sex?* *At school, college or university; At work (or through work); In a pub, bar, nightclub, or dance; Introduced by friends or family; At a faith group; Through a sports club or other organisation or society; On holiday or while travelling; Internet dating website/app; Had always known each other (e.g. as family friends or neighbours); In a public place (e.g. park, café, shop, public transport); They were a sex worker (prostitute/rent boy/male or female escort); Other please describe.*	MA7120	FPE7120	YPK5620
F5: The **very first time** you had sex: (Options: Yes, No, Don’t remember)			
F5a: Had you been drinking alcohol before it happened?	MA7130	FPE7130	YPK5630
F5b: Had you been using drugs before it happened?	MA7140	FPE7140	YPK5640
F5c: Was a condom used on this occasion?	MA7150	FPE7150	YPK5650
F5d: Was any other type of contraception/protection used?	MA7160	FPE7160	YPK5660
F5e: Did you regret having had this first sexual experience?	MA7170	FPE7170	YPK5670
F5e1: If yes, how much did you regret it? A bit; Quite a lot; Very much	MA7180	FPE7180	YPK5680
F6: Looking back at all your sexual experiences, is there anything you regret? No, no regrets; Regret some of it; Regret most of it; Regret all of it	MA7190	FPE7190	YPK5690
*F6a: Below are some reasons why you might have regrets: Please select all that apply.*			
F6a_1: Resulted in an unplanned pregnancy	MA7200	FPE7200	YPK5700
F6a_2: Resulted in a sexually transmitted infection	MA7210	FPE7210	YPK5710
F6a_3: Relationship was violent	MA7220	FPE7220	YPK5720
F6a_4: I felt I was being used	MA7230	FPE7230	YPK5730
F6a_5: I was using someone against their will	MA7240	FPE7240	YPK5740
F6a_6: Never found the right person	MA7250	FPE7250	YPK5750
F6a_7: It was against my religious faith	MA7260	FPE7260	YPK5760
F6a_8: It was too soon/we should have waited	MA7270	FPE7270	YPK5770
F6a_9: Had a termination/abortion	MA7280	FPE7280	YPK5780
F6a_10: I regret that I didn't have more sex	MA7290	FPE7290	YPK5790
F6a_99: Other, please describe	MA7300	FPE7300	YPK5800
*F7: The next questions are about the number of people you have had sex within your life and in the past 2 years. When answering these questions please include everyone you have had sex with, whether it was just once, a few times, or a regular partner. Options: 0; 1; 2–3; 4–9; 10+*			
F7a: Number of people within the last 24 months	MA7310	FPE7310	YPK5810
F7b: Number of people ever	MA7320	FPE7320	YPK5820
F8: How often are you having sex nowadays? Options: Not at all; Less than once a month; 1–3 times a month; About once a week; 2–4 times a week; 5 or more times a week.	MA7330	FPE7330	YPK5830
F9: In general, respondent do/did you enjoy sex? Options: Yes, very much; Yes, somewhat/sometimes; No, not a lot; No, not at all.	MA7340	FPE7340	YPK5840

a. Participants were asked about their sexual orientation and their level of attraction to men and women on a scale of 0 to 10. The latter is similar to a question asked in NATSAL-3, which had a scale of six attraction options.
b. We chose two items from each of three subscales from the Brief Sexual Attitudes Scale (BSAS) ( Hendrick *et al.*, 2006). The original scale comprised 23 items measuring four relatively independent sexual attitudes (we omitted birth control as this was covered elsewhere): “ *Listed below are several statements that reflect different attitudes about sex. For each statement, please say how much you agree or disagree with that statement*” on a 5-point Likert scale (5 = strongly agree to 1 = strongly disagree). These statements are used to describe three attributes or dimensions:

Permissiveness:

*Casual sex (e.g. a one-night stand) is acceptable.*

*It is okay to have ongoing sexual relationships with more than one person at a time.*

Communion (idealised sexuality):

*Sex is the closest form of communication between two people.*

*Sex is a very important part of life.*

Instrumentality (attitude of enjoyment of physical sex):

*The main purpose of sex is to enjoy oneself.*

*Sex is primarily a bodily function, like eating.*

The BSAS does not give an overall score, but rather scores for each attitude. Variables for each attitude were derived, giving a measure of each on a scale of 1–10, with lower scores equating to higher levels of that attitude. For example, the derived variable MA7042 is the dichotomised Permissiveness score (MA7041). A cut-point of 5 or less indicating high, and scores of 6–10 low permissiveness. MA7041 is the sum of variables MA7030 and MA7040, with data coded as missing if any responses are missing. The same methodology was used for the Communion and Instrumentality sub-scales, with scores 6+ equalling low levels of that attitude ( Hendrick & Hendrick, 1987; Hendrick *et al.*, 2006).

c. Two further statements in this section were aimed at eliciting opinions that could be heavily influenced by religious belief: “ *Sex should be reserved for marriage*” and “ *A central purpose of sex is to have children*”.
d. The next question asked the age at sexual debut (see Table 1 for exact wording) and then where they met their first sexual partner (adapted from NATSAL-3, as was the question on the total number of sexual partners within the last 2 years, and ever. Homepage | Natsal).
e. Questions on whether respondents had been drinking alcohol, using drugs, condoms and other contraception usage at their sexual debut were included. These questions were also asked in detail of the G1 cohort online at the Teen Focus clinics and in the questionnaires administered to them at ages 21 and 23 years (see Iles-Caven & Golding, 2024b).
f. Respondents were also asked if they regretted their sexual debut and the degree of regret; this was followed by a question on whether, looking back at all their sexual experiences, there was anything they regretted and reasons for that regret. They were encouraged to select all that applied (see Table 1 for options) and to describe any other reasons. These regret questions were developed from Kennair and colleagues’ (2018) work on sex differences in regret of casual sex. This paper found that women were more likely to regret (casual) sex due to disgust, worry (damage to reputation, STIs or pregnancy), feeling pressured and (sexual) competence of their partner. Men were more likely to regret passing up opportunities for casual sex. We also included an option relating to longer term relationships (i.e. the relationship was violent).

The ‘Other reason’ for regret was ticked by 18.3% mothers, 21.3% of partners, 24% of G1 females and 29.7% of G1 males, however, a free text response was not always provided. Responses ranged from a single word or term to a paragraph. Therefore, a number of responses fell into multiple categories and consequently some individuals may appear in up to four categories. Some of these categories have been collapsed to protect participants’ identity if numbers were small. The coding of these text responses is available on request.

g. Further questions asked about the number of sexual partners within the last 2 years and over the lifetime to date. The final two questions were devised by the authors as a positive ending to this section and asked how often the respondents were having sex nowadays and whether they generally enjoy/enjoyed sex.

### Observations

The frequencies in the tables are presented for the G0 mothers, their partners, and for G1 males and females (i.e. sex assigned at birth). It should be noted that the partners may or may not be the biological father, could change over time (< 5%) and could be of either sex (although <1% were female). Where cell counts are between 0–4, these are denoted as <5 in order to preserve confidentiality of the respondents. Here we use G0 mother, G0 partner and G1 males/females to differentiate the generations and sexes in the text.

Table 2a (G0) and Table 2b (G1) provide basic demographic details of the ALSPAC participants who responded to the 2022 questionnaires.

**Table 2a.** Basic demographics for the G0 (Parent) cohort who completed the 2022 questionnaire.

Demographic factor (variable name)	Mothers N	%	Partners N	%
	4653	100	1945	100
**Number of older brothers** *c450/pf2080*				
None	2581	55.5	462	23.8
1+	1735	37.3	522	26.8
**Number of older sisters** *c451/pf2081*				
None	2645	56.8	459	23.6
1+	1671	35.9	518	26.6
**Number of younger brothers** *c453/pf2082*				
None	2783	59.8	444	22.8
1+	1533	32.9	590	30.3
**Number of younger sisters** *c454/pf2083*				
None	2737	58.8	472	24.3
1+	1579	33.9	539	27.7
**Was a twin** *c456/pf2084*	75	1.6	27	1.4
**Age at delivery of G1** *mz028b; partner_age*				
<25	525	11.3	61	3.1
25–34	3270	70.3	1313	67.5
35+	594	12.8	562	28.9
**Ethnicity** *c800, c801*				
White	4213	90.5	1812	41.7
Other than white	84	1.8	29	1.5
**Highest Educ** *c645a, pb325a*				
<0 level	2141	46.0	577	29.7
A level+	2169	46.6	1142	58.7
**Marital status at enrolment** *a525, pa065*				
Married	3695	79.4	1491	76.7
Not married	694	14.9	148	7.6
**Social class** *c755, c765*				
Non-manual (I-IIINM)	3345	71.9	1344	69.1
Manual (IIIM-V)	468	10.1	421	21.6
**Housing tenure** *a006*				
Owner occupied	3737	80.3	-	-
Rented/all other	673	14.5		
**Urban/Rural** *Jan1993ur01ind_m*			-	-
Urban	3573	87.2		
Rural	524	12.8		
**IMD** *Jan2014imd2010q5*			-	-
1 least deprived	1736	40.6		
2	1124	26.3		
3	720	16.8		
4	466	10.9		
5 most deprived	231	5.4		

-Not available

**Table 2b.** Basic demographics of those G1 (Young People) who completed the 2022 questionnaire.

Demographic factor (variable name)	N	%
**Sex assigned at birth** (kz021)		
Female	2702	66.4
Male	1366	33.6
**First born**		
YP was the eldest child (kd431)	1652	47.1
YP was a member of twin birth (kb610)	86	2.4
**Self-reported ethnicity of YP** (DV YPH2012)		
White	3280	95.8
Other than white	142	4.2
**Highest Education level of YP (at age 26+)** (YPF7970)		
GCSE or equivalent	306	11.3
A level & higher NVQs	1074	39.5
Degree or higher	1337	49.2
**YP’s current relationship status (at age 29+)** (YPJ2500)		
Married	822	23.5
In civil partnership	75	2.1
Engaged	486	13.9
Other partner type (excl. any of above)	1343	38.3
No partner	777	22.2
**YP lives with partner (at age 29+)** (YPJ2510)		
Yes, all the time	2305	84.6
Yes, some of the time	159	5.8
No	261	9.6
**YP is currently a parent (age 29+)** (YPJ6000)		
Yes	914	26.3
No	2557	73.7

***Sexual orientation*.** Sexual orientation frequencies are given in Table 3. In G1, just over 80% of each sex described themselves as heterosexual compared with ~95% of the G0 cohort; almost 7% of G1 males described themselves as “homosexual” but only 1.5% of G1 females and <0.5% of the G0 mothers did so. The proportion describing themselves as “bisexual”, “pansexual” or “asexual” were higher in G1 females than G1 males and were much higher than that seen in the parent cohorts.

**Table 3.** Sexual orientation as reported in 2022 (N.B. Partners could be of either sex).

Sexual orientation	Mothers N (%)	Partners N (%)	YP Females N (%)	YP Males N (%)
***Variable name***	***MA7010***	***FPE7010***	***YPK5510***	
Gay/Lesbian	17 (0.4)	<5	41(1.5)	94 101 (6.9)
Bisexual/Pansexual/Asexual	55 (1.2)	32 (1.6)	292 (10.8)	100 (7.3)
Heterosexual/Straight	4394 (94.4)	1850 (95.1)	2204 (81.6)	1121 (82.1)
None of these/Prefer not to answer/Don’t know	78 (1.7)	20 (1.0)	73 (2.7)	23 (1.7)
Total *	**4653**	**1945**	**2702**	**1366**

*Totals are of returned questionnaires. Respondents may not have answered all sections or questions within the questionnaire. Some categories collapsed to protect participant identity: Gay/Lesbian; Bisexual + Pansexual + Asexual; None of these + Prefer not to answer + Don’t know.

Table 4 shows participants’ self-reported degree of attraction to each sex on a 10-point scale. Just over 47% of G1 females reported being attracted only to men compared with 60% of G1 males to only women. Of the G1 females, 2.1% reported to be equally attracted to both sexes compared with 0.7% of G1 males but 1.7% of G1 males and 3% of G1 females claimed that gender was not important to them. Interestingly, 1.2% of mothers and 1.1% of female G1s reported being attracted to only women; in comparison 1.8% of G0 partners vs. 5.1% of G1 males reported being attracted only to men.

**Table 4.** Degree of attraction to men and women (N.B. Partners could be of either sex). Cell counts <5 may equal zero.

Degree of attraction	Mothers N (%)	Partners N (%) ^#^	YP Females N (%)	YP Males N (%) ^##^
***Variable name***	***MA7020***	***FPE7020***	***YPK5520***	
0 Only men	3697 (79.4)	36 (1.8)	1279 (47.3)	70 (5.1)
1	206 (4.4)	6 (0.3)	382 (14.1)	29 (2.1)
2	146 (3.1)		349 (12.9)	6 (0.4)
3	69 (1.5)		228 (8.4)	11 (0.9)
4	37 (0.8)		82 (3.0)	
5 Equally to men and women	39 (0.8)	14 (0.7)	58 (2.1)	9 (0.7)
6	5 (0.1)	13 (0.7)	15 (0.6)	8 (0.6)
7	6 (0.1)		20 (0.7)	32 (2.3)
8	6 (0.1)	35 (1.8)	22 (0.8)	94 (6.9)
9	11 (0.2)	77 (4.0)	13 (0.5)	194 (14.2)
10 Only women	57 (1.2)	1651 (84.9)	30 (1.1)	827 (60.5)
Gender/sex is unimportant to me	38 (0.8)	7 (0.4)	80 (3.0)	23 (1.7)
Prefer not to answer	83 (1.8)	14 (0.7)	11 (0.4)	12 (0.9)
Don’t know	40 (0.9)	10 (0.5)	27 (0.1)	5 (0.4)
Total *	**4653**	**1945**	**2702**	**1366**

#For partners’ categories 1-4 and 6+7 collapsed to protect participant identity. ##For YP males’ categories 3+4 collapsed to protect participant identity. *Totals are of returned questionnaires. Respondents may not have answered all sections or questions within the questionnaire.

***Brief Sexual Attitudes Scale (BSAS)*.** Table 5a gives individual frequencies for each of the two questions from the Brief Sexual Attitudes Scale (BSAS) sub-scales relating to Permissiveness (reflects attitudes toward sexual permissiveness and openness), Communion (emotional intimacy and connection, idealised sexuality) and Instrumentality (pleasure, satisfaction). The scores for high permissiveness were over 45% for G1 females and over 58% for G1 males (mean for G1 males/females combined was 5.45), compared with ~11% of mothers and 18.7% of partners. For high communion almost identical frequencies were noted for the women in both generations (mothers 50.5%, G1 females 50.4%), with higher rates in the males (partners 67.8% and G1 males 60.2%). High instrumentality was found in ~50% of G1 compared with lower levels in the G0 generation (38.7% for mothers and 42.8% for their partners).

**Table 5a.** Brief Sexual Attitudes Scale: Degree to which respondent thinks certain behaviours are acceptable. Percentages are based on total response rate to the questionnaire, not the individual question and hence percentages will not = 100%.

	Mothers N (%)	Partners N (%)	YP Females N (%)	YP Males N (%)
Permissiveness				
*Casual sex is acceptable*	*MA7030*	*FPE7030*	*YPK5530*	
Strongly disagree	1432 (30.8)	545 (28.0)	902 (33.4)	605 (44.3)
Moderately disagree	787 (16.9)	283 (14.6)	742 (27.5)	373 (27.3)
Neither disagree nor agree	1379 (29.6)	472 (24.3)	542 (20.1)	189 (13.8)
Moderately agree	673 (14.5)	441 (22.7)	187 (6.9)	81 (5.9)
Strongly agree	225 (4.8)	158 (8.1)	235 (8.7)	78 (5.7)
*1+ concurrent sexual partners is acceptable*	*MA7040*	*FPE7040*	*YPK5540*	
Strongly disagree	2710 (58.2)	1026 (52.7)	307 (11.4)	232 (17.0)
Moderately disagree	833 (17.9)	315 (16.2)	468 (17.3)	281 (20.6)
Neither disagree nor agree	688 (14.8)	341 (17.5)	517 (19.1)	283 (20.7)
Moderately agree	179 (3.8)	154 (7.9)	533 (19.7)	263 (19.2)
Strongly agree	81 (1.7)	60 (3.1)	781 (28.9)	267 (19.5)
*Permissiveness score*	*MA7042*	*FPE7042*	*YPK5542*	
High permissiveness (0-5)	494 (10.6)	363 (18.7)	1218 (45.1)	796 (58.3)
Low permissiveness (6-10)	3992 (85.8)	1532 (78.8)	1387 (51.3)	530 (38.8)
Communion				
*Sex is the closest form of communication between two people*	*MA7050*	*FPE7050*	*YPK5550*	
Strongly disagree	684 (14.7)	182 (9.4)	266 (9.8)	168 (12.3)
Moderately disagree	739 (15.9)	265 (13.6)	652 (24.1)	330 (24.6)
Neither disagree nor agree	1030 (22.1)	427 (21.9)	581 (21.5)	370 (27.1)
Moderately agree	1171 (25.2)	573 (29.5)	721 (26.7)	308 (22.6)
Strongly agree	860 (18.5)	453 (23.3)	389 (14.4)	148 (10.8)
*Sex is a very important part of life*	*MA7060*	*FPE7060*	*YPK5560*	
Strongly disagree	352 (7.6)	68 (3.5)	632 (23.4)	450 (32.9)
Moderately disagree	522 (11.2)	131 (6.7)	1111 (41.1)	538 (39.4)
Neither disagree nor agree	1239 (26.6)	281 (14.4)	542 (20.0)	215 (15.7)
Moderately agree	1613 (34.7)	720 (37.0)	218 (8.1)	79 (5.8)
Strongly agree	768 (16.5)	697 (35.9)	103 (3.8)	44 (3.2)
*Communion score*	*MA7062*	*FPE7062*	*YPK5562*	
High communion (0-5)	2348 (50.5)	1318 (67.8)	1362 (50.4)	822 (60.2)
Low communion (6-10)	2127 (45.7)	578 (29.7)	1244 (46.0)	502 (36.7)
Instrumentality				
*Main purpose of sex is enjoyment*	*MA7070*	*FPE7070*	*YPK5570*	
Strongly disagree	213 (4.6)	110 (5.6)	681 (25.2)	294 (21.5)
Moderately disagree	496 (10.7)	237 (12.2)	1236 (45.7)	579 (42.4)
Neither disagree nor agree	1161 (24.9)	409 (21.0)	437 (16.2)	246 (18.0)
Moderately agree	1772 (38.1)	760 (39.1)	204 (7.6)	162 (11.9)
Strongly agree	838 (18.0)	382 (19.6)	49 (1.8)	45 (3.3)
*Sex is primarily a bodily function*	*MA7080*	*FPE7080*	*YPK5580*	
Strongly disagree	1026 (22.0)	418 (21.5)	120 (4.4)	110 (8.0)
Moderately disagree	997 (21.4)	435 (22.4)	521 (19.3)	351 (25.7)
Neither disagree nor agree	1413 (30.4)	458 (23.5)	810 (30.0)	367 (26.7)
Moderately agree	842 (18.1)	417 (21.4)	723 (26.8)	313 (22.9)
Strongly agree	193 (4.1)	167 (8.6)	430 (15.9)	185 (13.5)
*Instrumentality score*	*MA7082*	*FPE7082*	*YPK5582*	
High instrumentality (0-5)	1799 (38.7)	832 (42.8)	1327 (49.1)	696 (50.9)
Low instrumentality (6-10)	2662 (57.2)	1062 (54.6)	1276 (47.2)	630 (46.1)
Total *	**4653**	**1945**	**2702**	**1366**

* Totals are of returned questionnaires. Respondents may not have answered all sections or questions within the questionnaire.

**Table 5b.** Frequencies for the non-BSAS questions.

Non-BSAS questions	Mothers N (%)	Partners N (%)	YP Females N (%)	YP Males N (%)
*Sex should be reserved for marriage*	*MA7090*	*FPE7090*	*YPK5590*	
Strongly disagree	2413 (51.9)	936 (48.1)	1993 (73.8)	1046 (76.6)
Moderately disagree	739 (15.9)	359 (18.6)	306 (11.3)	149 (10.9)
Neither disagree nor agree	835 (17.9)	331 (17.0)	200 (7.4)	72 (5.3)
Moderately agree	244 (5.2)	125 (6.4)	42 (1.5)	22 (1.6)
Strongly agree	250 (5.4)	143 (7.3)	66 (2.4)	37 (2.7)
*Main purpose of sex is to have children*	*MA7100*	*FPE7100*	*YPK5600*	
Strongly disagree	2087 (44.9)	712 (36.6)	1215 (45.0)	565 (41.4)
Moderately disagree	786 (16.9)	384 (19.7)	500 (18.5)	254 (18.6)
Neither disagree nor agree	982 (21.1)	434 (22.3)	475 (17.6)	275 (20.1)
Moderately agree	493 (10.6)	256 (13.2)	347 (12.8)	193 (14.1)
Strongly agree	125 (2.7)	108 (5.5)	65 (2.4)	39 (2.8)
Total *	**4653**	**1945**	**2702**	**1366**

*Totals are of returned questionnaires. Respondents may not have answered all sections or questions within the questionnaire.

Similar proportions of both sexes endorsed moderate/strong agreement that sex should be reserved for marriage (3.9% of G1 females and 4.3% of G1 males) compared with 10.6% of mothers and 13.7% of partners). Only 2.4% of female and 2.8% of male G1s strongly agreed that the main purpose of sex was to have children, and this was very similar to mothers’ attitudes (2.7%), but a larger proportion of partners endorsed this view (5.5%).

Table 6a– Table 6e give the correlation coefficients for the items reported in Table 5a and Table 5b, along with the p values. The correlation coefficients at p<0.0001 ranged from -0.13 to 0.55 for the G0 mothers, from -0.38 to 0.60 for the G0 fathers and -0.12 to 0.62 for the G1s. Unsurprisingly, each pair of questions measuring the three attribute scores (permissiveness, communion and instrumentality) were highly correlated, the highest correlation coefficients for all three cohorts being for the relationships between the two permissiveness items: casual sex is OK, and sex with more than one person at a time is permissible.

**Table:** 

Key for Tables 6a–6e:
*Brief Sexual Attitudes Scale questions*
Permissiveness
MA7030/YPK5530 – Casual sex is acceptable
MA7040/YPK5540 – 1+ concurrent partners is acceptable
Communion
MA7050/YPK5550 – Sex is closest form of communication
MA7060/YPK5560 – Sex us a very important part of life
Instrumentality
MA7070/YPK5570 – Primary point of sex is enjoyment
MA7080/YPK5580 – Sex is bodily function
*Non-BSAS items likely to score highly in religious groups*
MA7090/YPK5590 – Sex should be reserved for marriage
MA7100/YPK5600 – Central purpose of sex is to have children

**Table 6a.** Correlation coefficients with *p* values (bold indicates *p*<.0001) Mothers Brief Sexual Attitudes Scale.

Mother	MA7030	MA7040	MA7050	MA7060	MA7070	MA7080	MA7090	MA7100
**MA7030**	1.00							
**MA7040**	**0.55**	1.00						
**MA7050**	**-0.10**	**-0.07**	1.00					
**MA7060**	0.04 .009	0.04 .010	**0.42**	1.00				
**MA7070**	**0.12**	**0.07**	**0.21**	**0.28**	1.00			
**MA7080**	**0.12**	**0.13**	**0.13**	**0.09**	**0.19**	1.00		
**MA7090**	**0.36**	**0.18**	**-0.13**	0.01 .407	**0.14**	0.01 .420	1.00	
**MA7100**	**0.15**	**0.09**	**-0.07**	0.05 .001	**0.12**	**-0.12**	**0.42**	1.00

**Table 6b.** Correlation coefficients with *p* values (bold indicates *p*<.0001) for Partners Brief Sexual Attitudes Scale.

Partner	FPE7030	FPE7040	FPE7050	FPE7060	FPE7070	FPE7080	FPE7090	FPE7100
**FPE7030**	1.00							
**FPE7040**	**0.60**	1.00						
**FPE7050**	-0.09 .001	-0.06 .011	1.00					
**FPE7060**	0.06 .010	0.07 .003	**0.40**	1.00				
**FPE7070**	**0.11**	**0.13**	**0.14**	**0.21**	1.00			
**FPE7080**	**0.14**	**0.17**	0.02 .371	0.01 .715	**0.27**	1.00		
**FPE7090**	**-0.38**	**-0.23**	0.07 .001	-0.08 .001	**-0.11**	-0.03 .265	1.00	
**FPE7100**	**-0.09**	-0.06 .010	0.00 .830	-0.02 .335	-0.02 .314	**0.11**	**0.35**	1.00

**Table 6c.** Correlation coefficients with *p* values (bold indicates *p*<.0001) all G1s Brief Sexual Attitudes Scale.

G1s	YPK5530	YPK5540	YPK5550	YPK5560	YPK5570	YPK5580	YPK5590	YPK5600
**YPK5530**	1.00							
**YPK5540**	**0.62**	1.00						
**YPK5550**	**-0.11**	**-0.12**	1.00					
**YPK5560**	**0.21**	**0.13**	**0.34**	1.00				
**YPK5570**	**0.23**	**0.15**	**0.13**	**0.27**	1.00			
**YPK5580**	**0.11**	**0.15**	**0.16**	**0.21**	**0.22**	1.00		
**YPK5590**	**0.45**	**0.27**	**-0.08**	**0.13**	**0.21**	**0.06**	1.00	
**YPK5600**	**0.24**	**0.19**	**-0.08**	0.05 .001	**0.10**	-0.06 .001	**0.33**	1.00

**Table 6d.** Correlation coefficients with *p* values (bold indicates *p*<.0001) G1 Males Brief Sexual Attitudes Scale.

G1 Male	YPK5530	YPK5540	YPK5550	YPK5560	YPK5570	YPK5580	YPK5590	YPK5600
**YPK5530**	1.00							
**YPK5540**	**0.61**	1.00						
**YPK5550**	-0.10 .001	-0.09 .001	1.00					
**YPK5560**	**0.23**	**0.14**	**0.32**	1.00				
**YPK5570**	**0.23**	**0.16**	**0.13**	**0.24**	1.00			
**YPK5580**	0.10 .001	**0.14**	**0.15**	**0.17**	**0.27**	1.00		
**YPK5590**	**0.53**	**0.31**	-0.10 .0001	**0.13**	**0.20**	0.06 .026	1.00	
**YPK5600**	**0.23**	**0.17**	-0.07 .010	0.04 .139	0.06 .034	-0.09 .001	**0.32**	1.00

**Table 6e.** Correlation coefficients with *p* values (bold indicates *p*<.0001) for G1 Females Brief Sexual Attitudes Scale.

G1 female	YPK5530	YPK5540	YPK5550	YPK5560	YPK5570	YPK5580	YPK5590	YPK5600
**YPK5530**	1.00							
**YPK5540**	**0.62**	1.00						
**YPK5550**	**-0.12**	**-0.14**	1.00					
**YPK5560**	**0.19**	**0.12**	**0.34**	1.00				
**YPK5570**	**0.24**	**0.16**	**0.14**	**0.30**	1.00			
**YPK5580**	**0.11**	**0.14**	**0.16**	**0.22**	**0.21**	1.00		
**YPK5590**	**0.42**	**0.24**	-0.07 .001	**0.13**	**0.21**	**0.05**	1.00	
**YPK5600**	**0.26**	**0.21**	-0.08 .001	0.07 .001	**0.13**	.073 -0.04	**0.33**	1.00

***Risky Behaviours*.** Table 7 summarises the responses to the questions on ‘risky’ behaviours. By the age of ~30 years for the G1 cohort, 2.4% of females and 4.9% of males reported they had not yet experienced sexual intercourse. Females were more likely than males to report sexual debut prior to 16 years of age: 30.6% of females were aged 15 and under compared with 19.2% of males. However, the contrast between the female generations is quite marked with 13.7% of G0 mothers reporting sexual debut before the age of 16. A difference was seen in the male generations, but it was not as wide (11% in G0 partners compared to 18.7% of G1 males).

**Table 7.** Frequencies relating to risky sexual behaviours. <5 may equal zero. Percentages are of total respondents to questionnaires for the parents, but only of the sexually active offspring.

	Mothers N (%)	Partners N (%)	YP Females N (%)	YP Males N (%)
Has not had sex yet (as % of total respondents)	N/A	N/A	65 (2.4)	67 (4.9)
*Age at sexual debut*	*MA7110*	*FPE7110*	*YPK5610*	
≤12 years	25 (0.5)	11 (0.6)	16 (0.6)	7 (0.5)
13	54 (1.2)	18 (0.9)	67 (2.5)	23 (1.8)
14	162 (3.5)	65 (3.3)	223 (8.5)	75 (5.8)
15	401 (8.6)	127 (6.5)	500 (19.0)	144 (11.1)
16	874 (18.8)	272 (14.0)	561 (21.3)	260 (20.0)
17–20	2226 (47.8)	948 (48.7)	935 (35.5)	552 (42.5)
21–24	392 (8.4)	253 (13.0)	119 (4.5)	113 (8.7)
25+ years	122 (2.6)	114 (5.7)	50 (1.9)	34 (2.6)
Prefer not to answer	257 (5.5)	90 (4.6)	69 (2.6)	44 (3.4)
*Met first sexual partner at:*	*MA7120*	*FPE7120*	*YPK620*	
School, College, University	1475 (31.7)	714 (36.7)	1232 (46.7)	651 (50.1)
At or through work	394 (8.5)	183 (9.4)	133 (5.0)	53 (4.1)
Pub, bar, night club, dance	786 (16.9)	283 (14.5)	149 (5.6)	82 (0.1)
Introduced by family, friends	903 (19.4)	289 (14.9)	559 (21.2)	193 (14.9)
Sports club or other organisation/society	136 (2.9)	81 (4.2)	17 (0.6)	12 (0.9)
Faith group	239 (5.1)	108 (5.6)	106 (4.0)	63 (4.8)
On holiday or while travelling	167 (3.6)	74 (3.8)	78 (3.0)	37 (2.8)
Internet dating website/app	12 (0.3)	<5	105 (4.0)	86 (6.6)
Always known each other	103 (2.2)	47 (2.4)	39 (1.5)	20 (1.5)
Public place (park, café, shop, public transport)	110 (2.4)	35 (1.8)	65 (2.5)	15 (1.1)
Other	122 (2.6)	56 (3.3)	43 (1.6)	39 (3.0)
*Had been drinking alcohol prior to sexual debut*	*MA7130*	*FPE7130*	*YPK5630*	
No	3182 (68.4)	1232 (63.3)	1686 (63.9)	791 (60.9)
Yes	645 (13.9)	427 (21.9)	732 (27.8)	390 (30.0)
Cannot remember	606 (13.0)	224 (11.5)	114 (4.3)	71 (5.5)
*Had been using drugs prior to sexual debut*	*MA7140*	*FPE7140*	*YPK5640*	
No	4354 (93.6)	1839 (94.5)	2459 (93.2)	1194 (91.9)
Yes	19 (0.4)	7 (0.4)	39 (1.5)	26 (2.0)
Cannot remember	40 (0.9)	24 (1.2)	26 (0.1)	28 (2.1)
*Used a condom at sexual debut*	*MA7150*	*FPE7150*	*YPK5650*	
No	1847 (39.7)	1058 (54.4)	533 (20.2)	323 (24.9)
Yes	2157 (46.4)	711 (36.6)	1843 (69.9)	862 (68.7)
Cannot remember	424 (9.6)	111 (5.7)	156 (5.9)	63 (4.8)
*Used other type of contraception at sexual debut*	*MA7160*	*FPE7160*	*YPK5660*	
No	2603 (60.4)	1053 (54.1)	1516 (57.5)	679 (52.3)
Yes	1293 (30.0)	553 (28.4)	889 (33.7)	364 (28.0)
Cannot remember	413 (9.1)	246 (12.6)	120 (4.6)	199 (15.3)
*No. sexual partners in previous 2 years*	*MA7310*	*FPE7310*	*YPK5810*	
0	1206 (25.9)	369 (19.0)	135 (5.1)	71 (5.4)
1	3116 (67.0)	1440 (74.0)	2009 (76.2)	897 (69.0)
2–3	81 (1.7)	49 (2.5)	251 (9.5)	158 (12.2)
4–9	9 (0.2)	8 (0.4)	121 (4.6)	71 (5.5)
10+	<5	6 (0.3)	16 (0.6)	47 (3.6)
*Total no. sexual partners in life*	*MA7320*	*FPE7320*	*YPK5820*	
1	924 (19.9)	352 (18.1)	291 (11.0)	153 (11.8)
2–3	1121 (24.1)	394 (20.3)	396 (15.0)	238 (18.3)
4–9	1462 (31.4)	624 (32.1)	801 (30.4)	358 (27.6)
10+	811 (17.4)	455 (23.4)	1011 (38.3)	483 (37.9)
Total*	**4653**	**1945**	**2637**	**1299**

Generational differences were also evident for where participants met their first sexual partner: A greater proportion of G1s met at an educational institution or via an internet dating website/app compared to G0s. Conversely, G0s were more likely to have met through work; at a pub, bar, nightclub, dance/disco; a sports club or other organisation. For both generations and sexes, an educational institution (school, college or university) was the most frequently cited followed by being introduced by family or friends ( Table 7).

Whilst very few participants in both generations reported using drugs prior to their sexual debut (<2%), alcohol had been consumed by around a third of G1s, compared to 14% of G0 mothers and 22% of G0 partners. Perhaps unsurprisingly around double the older generation (over 10%) stated they could not remember ( Table 7).

Condom use at sexual debut is one measure of ‘sexual readiness’ and almost 70% of G1 recalled using a condom, compared with 46.4% of G0 mothers and 36.6% of G0 partners. Between 28% and 34% of all participants recalled using another type of contraception at sexual debut, but these were not specified in this questionnaire ( Table 7). Detailed information on types of contraception used have been collected previously at multiple time points.

The majority of G0s (67% mothers vs. 74% of partners) reported having had just one sex partner within the previous two years; this is comparable to G1 females (76.2%) and 69% of G1 males. Six-times as many G1 males reported 10 or more partners (3.6%) vs 0.6% of G1 females. Just over 5% of G1s reported zero partners in the last 2 years, compared with much higher rates in G0s (almost 26% of mothers and 19% of the partners) ( Table 7).

Perhaps unsurprisingly there were generational and sex differences regarding the number of sexual partners over the lifespan: roughly similar proportions of mothers and partners reported one partner, 2-3 or 4-9 partners. The largest differences between the generations were seen in those reporting one sexual partner with ~11% of G1s and ~20% of G0s reporting this; and the 10+ sexual partners category with 38.3% of G1 females vs 17.4% of G0 mothers and 37.9% of G1 males vs 23.4% of the G0 partners ( Table 7).

***Frequency and Enjoyment of Sexual Intercourse*.** Table 8 summarises the responses to current frequency and enjoyment of sexual intercourse. In the G1s, similar proportions of both sexes are seen for all six options for frequency. Over a third of mothers and 26.2% of partners reported no sex at all. G0 reported reduced frequencies overall compared to G1. The most striking differences between the sexes was in the proportion of participants who reported that they enjoyed sexual intercourse. A small proportion of respondents reported that they did not enjoy sex at all. More women in both generations stated ‘No, not a lot’ (7.7% of G0 mothers and 4.4% of their G1 daughters vs. 1.4% of G0 partners <1% of the G1 sons). 40% of G0 mothers and 48% of G1 daughters reported they enjoyed sex “very much” compared to 75.5% of G0 partners and 71.2% of G1 sons ( Table 8).

**Table 8.** Reported frequencies for sexual intercourse and of enjoyment of sex. <5 may equal zero. Percentages are of total respondents to questionnaires for the parents, but only of sexually active offspring.

	Mothers N (%)	Partners N (%)	YP Females N (%)	YP Males N (%)
*Frequency of sexual intercourse nowadays*	*MA7330*	*FPE7330*	*YPK5830*	
Not at all	1595 (34.3)	510 (26.2)	270 (10.2)	111 (8.5)
<1 month	1039 (22.3)	529 (27.2)	453 (17.2)	247 (19.0)
1–3 times/month	880 (18.9)	441 (22.7)	735 (27.9)	340 (26.2)
About once/week	594 (12.8)	264 (13.6)	622 (23.6)	303 (23.3)
2–4 times/week	297 (6.4)	126 (6.5)	411 (15.6)	222 (17.1)
5 or more times/week	23 (0.5)	8 (0.4)	41 (1.6)	22 (1.7)
*Generally, respondent enjoys sex*	*MA7340*	*FPE7340*	*YPK5840*	
Not at all	73 (1.7)	7 (0.4)	17 (0.6)	<5
No, not a lot	342 (7.3)	27 (1.4)	116 (4.4)	22 (1.7)
Yes, somewhat/sometimes	2243 (48.2)	427 (22.7)	1139 (43.2)	298 (22.9)
Yes, very much	1769 (38.0)	1419 (75.5)	1262 (47.9)	925 (71.2)
**Total (100%)**	**4653**	**1945**	**2637**	**1299**

***Sexual Regret*.** Table 9a provides the frequencies related to sexual regret. Women were more likely to have sexual regrets than men. Interestingly, both sexes in the younger generation regretted their sexual debut slightly more than the parent generation. Most striking is the frequencies of those reporting regretting their sexual debut ‘quite a lot’ or ‘very much’: 10% of G0 mothers and G1 daughters compared to 2.7% of G0 partners and 3.5% of G1 males.

**Table 9a.** Frequencies related to sexual regret. Please note that participants were asked to tick all the reasons for regret that applied or may not have ticked any. Cell counts of <5 may include zero.

Percentages of total respondents to Qs	Mothers N=4653 (%)	Partners N=1945 (%)	YP Females N=2637 (%)	YP Males N=1299 (%)
*Respondent regretted their sexual debut*	*MA7170*	*FPE7170*	*YPK5670*	
No	3520 (75.6)	1721 (88.5)	1960 (74.3)	1099 (84.6)
Yes	779 (16.7)	109 (5.6)	541 (20.5)	124 (9.5)
Cannot remember	126 (2.7)	45 (2.3)	31 (1.2)	24 (1.8)
*Degree to which the respondent regretted their sexual debut*	*MA7180*	*FPE7180*	*YPK5680*	
A little bit	279 (5.6)	55 (2.8)	208 (7.9)	77 (5.9)
Quite a lot	278 (5.6)	33 (1.7)	160 (6.1)	22 (1.7)
Very much	207 (4.5)	20 (1.0)	170 (6.4)	24 (1.8)
*When respondent looks back at all their sexual experiences, they have regrets*	*MA7190*	*FPE7190*	*YPK5690*	
No, no regrets	2687 (57.7)	1366 (70.2)	1120 (44.5)	775 (59.7)
Regret some of it	1618 (34.8)	501 (25.8)	1279 (48.5)	461 (35.5)
Regret most of it	101 (2.2)	7 (0.4)	114 (4.3)	16 (1.2)
Regret all of it	30 (0.6)	7 (0.4)	20 (0.8)	<5
*Of those with regrets, reasons for regret if indicated (multiple items may have been ticked):*	N (%)	N (%)	N (%)	N (%)
Resulted in unplanned pregnancy *	*MA7200*	*FPE7200*	*YPK5700*	
Yes	228 (13.6)	52 (10.6)	148 (10.5)	32 (6.7)
*Resulted in sexually transmitted infection*	*MA7210*	*FPE7210*	*YPK5710*	
Yes	141 (8.4)	44 (9.0)	259 (18.3)	81 (16.9)
*The relationship was violent*	*MA7220*	*FPE7220*	*YPK5720*	
Yes	131 (7.8)	<5	166 (11.7)	13 (2.7)
*Respondent felt they were being used*	*MA7230*	*FPE7230*	*YPK5730*	
Yes	641 (38.2)	27 (5.5)	697 (49.3)	87 (18.2)
*Respondent used someone against their will*	*MA7240*	*FPE7240*	*YPK5740*	
Yes	5 (0.3)	9 (1.8)	7 (0.5)	8 (1.7)
*Never found the right person*	*MA7250*	*FPE7250*	*YPK5750*	
Yes	173 (10.3)	54 (11.0)	245 (17.3)	88 (18.4)
*Against their religious faith*	*MA7260*	*FPE7260*	*YPK5760*	
Yes	66 (3.9)	17 (3.5)	25 (1.8)	8 (1.7)
*It happened too soon/they should have waited*	*MA7270*	*FPE7270*	*YPK5770*	
Yes	459 (27.4)	69 (14.1)	412 (29.2)	73 (15.3)
Resulted in a termination/abortion *	*MA7280*	*FPE7280*	*YPK5780*	
Yes	172 (10.3)	14 (2.9)	143 (10.1)	19 (4.0)
*Regrets they didn’t have more sex*	*MA7290*	*FPE7290*	*YPK5790*	
Yes	142 (8.5)	203 (41.3)	143 (10.1)	162 (33.9)
*Other reasons for regret*	*MA7300*	*FPE7300*	*YPK5800*	
Yes	307 (18.3)	105 (21.3)	344 (24.3)	142 (29.7)

*The ‘mother’ may not have informed her sex partner of the pregnancy; terminations of pregnancy may also be included under unplanned pregnancies.

44% of G1 females reported that they had no regrets at all (compared with almost 60% of their G0 mothers). Almost half of these young G1 women said they regretted some and over 5% claimed to regret most or all of their sexual experiences. All levels of regret were much higher in the G1 females compared to all other groups.

Of those that expressed any level of regret, differences can be seen between the generations: slightly fewer unplanned pregnancies were reported in the G1s; and similar rates in both generations when it came to such a pregnancy ending in a termination. A large difference was seen inter-generationally in contracting a sexually transmitted infection (STI), where frequencies were roughly double in the G1s. Sex differences were noted with women in both generations more likely to report regret due to a violent relationship, or that they felt they were being used (both reasons slightly higher in the G1 females compared G0 mothers). However, G1 males were more likely to regret a violent relationship or feel they were being used compared with G0 partners. Not finding the “right person” was almost twice as common among G1s compared with G0s, but this probably reflects their youth ( Table 9a).

Women in both generations were much more likely to report regret because sex happened too soon/they should have waited (again roughly double than male responders). Conversely, both G0 partners and G1 males were more likely to express regret that they had not had more sex (41% and 34% respectively) versus G0 mothers (8.5%) and G1 females (10.1%) ( Table 9a).

Table 9b summarises the free text responses to ‘other reason’ for regret. Participants were most likely to report regretting unfaithfulness, ‘promiscuity’, immaturity or the wrong person/reason etc. Of particular note, was the much higher frequencies of experiencing sexual abuse/rape/non-consensual sex in women in both generations. In addition, women were more likely to report that a specific sexual encounter would not have happened if they had had the confidence to say ‘no’, i.e. acquiescence. Please note that these text responses have not yet been mapped to the tick box responses in Table 9a and there may few duplicates where they were giving more detail to a ticked box.

**Table 9b.** Summarisation of the most common other reasons (text responses) for regretting their sexual past (these have not been compared with tick box answers in Table 9a; individuals may appear under more than one reason; some may have ticked other reason but omitted to add text). Cell counts of <5 may include zero.

Other reasons for regret (individuals may appear under 1+ categories)	Mothers N (%)	Partners N (%)	YP Females N (%)	YP Males N (%)
*Variable name*	*MA7300*	*FPE7300*	*YPK5800*	
**Total who ticked ‘other’ regardless of whether or not a text response was given**	**307**	**105**	**344**	**142**
Being unfaithful to own partner/Discovered sex partner was being unfaithful to their spouse/partner	33	14	17	12
Historical sexual abuse/sexual assault of self or partner; rape; IPV; specified non-consensual sex whilst drunk; I was coerced/I was coercive; Physical or emotional abuse	44	<5	63	8
Ruined friendship(s); Slept with work colleague	<5	<5	8	8
Too casual/impulsive; too promiscuous; meaningless sex; A one-night stand; thoughtlessness; selfishness; paid for sex	51	20	29	10
Caused later emotional problems incl. hurting others; loss of self-confidence/self-esteem; induced anxiety. Unrequited love; to boost self-esteem; to feel happy; to give me confidence; to cure loneliness, insecurity. Guilt NOS.	9	7	24	9
Immaturity/Inexperience (incl. performance issues, lack of enjoyment, we should have waited), embarrassment.	23	16	21	13
Wrong person/time/location/reason incl. rebound & revenge sex. Problem(s) with partner’s attributes.	53	14	62	27
More adventurous/not enough sex/missed opportunities/should have experimented more. Just some regrets NOS	11	<5	<5	6
Did not use protection or resulted in pregnancy *; Worries/anxiety about the risk of pregnancy or catching an STI; Putting oneself in physical danger.	6	<5	12	11
Alcohol/Drunkenness/Drugs without the label ‘non-consensual’, e.g. we were both too drunk; ‘beer-goggles’.	6	<5	40	18
Peer pressure; It was expected; Not saying ‘no’, Not having the confidence to say ‘no’; acquiescence	13	<5	22	<5
Caused other complications e.g. awkwardness	10	<5	15	6
Unclassifiable (e.g. private, prefer not to say, cannot remember)	14	6	7	9

IPV = Intimate Partner Violence; NOS = Not otherwise stated; STI = sexually transmitted infection *Whether or not pregnancy resulted in a termination or livebirth.

### Strengths and limitations

A key strength of ALSPAC is that the population is geographically defined and included the majority (~80%) of the eligible pregnant population at the time of enrolment. Other strengths include a large sample size, the longitudinal design, regular administration of questionnaires with repeated questions over time, genetic and other -omics data, and annual face-to-face clinics for the offspring that reduce the chance of recall error in the earlier sweeps on sexual experiences (and other data), which will likely be used in conjunction with these 2022 data, plus the availability of data on potential confounding factors.

Extensive data collection in ALSPAC on sexual experiences, sexual functioning, sexually transmitted infections (STIs), sexual well-being and behaviour in previous sweeps allows for research on a wide variety of topics. The 2022 data sweep allows comparison of general attitudes towards sex, with current enjoyment and regret of their sexual experiences. As identical data has been collected on both generations this allows for intergenerational comparisons. Some questions were identical to those asked by NATSAL-3 which allows for direct comparisons. There is, however, one caveat to this: the initially enrolled mothers and their partners were soon to be parents and thus not generalisable to the non-reproducing population.

As with all longitudinal studies, attrition in ALSPAC is strongly related to socioeconomic position. ALSPAC participants were more likely to become lost to follow-up in the early years if they had experienced greater adversity during the index pregnancy (e.g. lack of social support or inadequate housing). Therefore, selection bias becomes a problem such that the current participants are less socio-economically deprived and have higher educational attainment compared with those no longer taking part. Analyses of the ways in which such attrition may bias results has been shown to be minimal ( Morgan *et al.*, 2022).

Both G0 and G1 males are more likely to drop out than G0 and G1 female participants. At the time of enrolment there were very few families of other than White ethnicity in the Avon area (~6%) and therefore research results cannot necessarily be extrapolated to non-White populations in general.

Since these data were self-reported they may be subject to recall error (particularly for G0s), if the experiences described occurred many years ago and therefore some events may have been forgotten or misremembered. It is also possible that respondents who had traumatic initial sexual experiences may have mentally blocked them out (e.g. Carver *et al.*, 1989; Lyon, 2007). Retrospective reporting may lead to possible recall bias (for example, if participants believed that an earlier sexual experience was a cause of their past or current mental health problems, see Das & Otis, 2016, for a review of the literature; and Liu *et al.* (2022)).

The questions on sexual experiences, attitudes, enjoyment and regret are sensitive, and some respondents chose not to answer them. 1.9% of mothers who returned the questionnaire missed the entire section; for partners 0.97% missed the section. for G1s, 2.87% missed the section (83 females (3.07%) and 34 (2.47%) males). It is possible that those with high numbers of sexual partners omitted this section due to social desirability or did not want to ‘relive’ traumatic experiences or simply felt it was too private to share. Although some respondents may have felt some kind of pressure to answer questions in a socially acceptable way, ALSPAC participants have previously answered sensitive questions (e.g. on adverse experiences and mental health) safe in the knowledge that confidentiality is of paramount importance to the study. For example, in an antenatal questionnaire to the mothers with a section on childhood sexual experiences and abuse: that section was omitted by only 10% of the mothers (see Iles-Caven & Golding, 2024a).

## Conclusion

This rich data resource may be of interest to researchers examining the transgenerational differences in sexual attitudes and behaviours, age at sexual debut, numbers of sexual partners, potential effects on health and well-being, and the quality of intimate relationships.

## Ethics and consent

Ethical approval for the ALSPAC study was obtained from the ALSPAC Ethics and Law Committee (ALEC; IRB00003312) and the Local Research Ethics Committees. Detailed information on the ways in which confidentiality of the cohort is maintained may be found on the study website: http://www.bristol.ac.uk/alspac/researchers/research-ethics/. All methods were performed in accordance with the relevant guidelines and regulations . Informed written consent for the use of data collected through questionnaires and clinics was provided by participants following the recommendations of the ALSPAC Ethics and Law Committee at the time. Study participants have the right to withdraw their consent for elements of the study or from the study entirely at any time ( Birmingham, 2018).

## Data Availability

ALSPAC data access is through a system of managed open access. The steps below highlight how to apply for access to the data included in this paper and all other ALSPAC data. Note that variable names are included in the tables within this paper. Please read the ALSPAC access policy ( http://www.bristol.ac.uk/media-library/sites/alspac/documents/researchers/data-access/ALSPAC_Access_Policy.pdf) which describes the process of accessing the data and biological samples in detail, and outlines the costs associated with doing so. You may also find it useful to browse our fully searchable research proposals database ( https://proposals.epi.bristol.ac.uk/), which lists all research projects that have been approved since April 2011. Please submit your research proposal ( https://proposals.epi.bristol.ac.uk/) for consideration by the ALSPAC Executive Committee using the online process. You will receive a response within 10 working days to advise you whether your proposal has been approved. If you have any questions about accessing data, please email: alspac-data@bristol.ac.uk (data) or bbl-info@bristol.ac.uk (samples). The ALSPAC data management plan ( http://www.bristol.ac.uk/media-library/sites/alspac/documents/researchers/data-access/alspac-data-management-plan.pdf) describes in detail the policy regarding data sharing, which is through a system of managed open access.
