# Supplementary material for: Sexual experiences, attitudes, enjoyment and regret in the parent and offspring generations of the Avon Longitudinal Study of Parents and Childhood (ALSPAC): 2022 data sweep
Source: Wellcome Open Res. 2026 Apr 14;9:674. Originally published 2024 Nov 12. [Version 2] doi: 10.12688/wellcomeopenres.23059.2 (PMC13090757; doi:10.12688/wellcomeopenres.23059.2)
Supplement: Peer review history

# Reviewer report: Reviewer response for version 2

**DOI:** 10.21956/wellcomeopenres.29042.r153201

**Published:** 2026-04-17

**Authors:** Chantal den Daas

**Affiliations:** 1 University of Aberdeen, Aberdeen, UK

The authors have carefully and thoughtfully replied to all observations. I have no other observations.

Are sufficient details of methods and materials provided to allow replication by others?

No

Is the rationale for creating the dataset(s) clearly described?

Yes

Are the datasets clearly presented in a useable and accessible format?

Yes

Are the protocols appropriate and is the work technically sound?

Yes

Reviewer Expertise:

Health psychology, behaviour in infection prevention, focus on sexual health.

I confirm that I have read this submission and believe that I have an appropriate level of expertise to confirm that it is of an acceptable scientific standard.

# Reviewer report: Reviewer response for version 1

**DOI:** 10.21956/wellcomeopenres.25395.r140049

**Published:** 2025-12-31

**Authors:** Elise Bragard

**Affiliations:** 1 Psychological and Brain Sciences, Fairfield University College of Arts and Sciences, Fairfield, Connecticut, USA; 2 University of Connecticut School of Medicine, Farmington, Connecticut, USA

This data note describes the methods and results of a follow-up questionnaire administered to participants in the ALSPAC (both original mothers and partners, and offspring). This questionnaire focuses on sexual attitudes and sexual regret, and the goal of the current analyses is to highlight intergenerational similarities and differences (overall and by sex) and sex differences. 

 The description of the questionnaire items, instructions, variable names, and coding instructions is extremely clear, and allows for replicability and reproducibility of the study. 

 Descriptive statistics for demographic items and the BSA scale are clearly reported in tables and in the text. Correlations across items/scales are clearly reported overall and by subgroup. Generational and sex differences are highlighted.

 Study limitations are comprehensive and clearly described. 

 The overall goal of the data note is to support future researchers who wish to use the open-access data for further research and this goal has been achieved.

Are sufficient details of methods and materials provided to allow replication by others?

Yes

Is the rationale for creating the dataset(s) clearly described?

Yes

Are the datasets clearly presented in a useable and accessible format?

Yes

Are the protocols appropriate and is the work technically sound?

Yes

Reviewer Expertise:

Developmental psychology, sexual development, sexual victimization, and substance use

I confirm that I have read this submission and believe that I have an appropriate level of expertise to confirm that it is of an acceptable scientific standard.

# Response

**Published:** 2026-03-26

**Authors:** Yasmin Iles-Caven

**Affiliations:** Bristol Medical School: Population Health Sciences, University of Bristol, Bristol, UK

We thank you for your comments on our paper.

# Reviewer report: Reviewer response for version 1

**DOI:** 10.21956/wellcomeopenres.25395.r117130

**Published:** 2025-02-06

**Authors:** Chantal den Daas

**Affiliations:** 1 University of Aberdeen, Aberdeen, UK

Thank you for the opportunity to review this data note, which describes data in the Avon Longitudinal Study of Parents and Childhood (ALSPAC) collected in 2022 specifically addressing questions around sexual behaviours and associated attitudes, regrets and enjoyment. It is a beautiful cohort, and having multiple generations is a real strength, moreover, having very interesting questions posed in a similar way to other big studies such as Natsal and posed in the same way to both generations, offer a lot of opportunities for research. Below some questions and comments, more than expected but this is because I was interested in the data.

 Background:

- Early debut or early activity could benefit from a definition here, what is early, or what is too early?
- I struck me that the background was slightly negative. In recent years we see that the younger generations delay initiation of sexual intercourse (see for example https://doi.org/10.1080/00224499.2023.2297906). I am not sure the introduction captures  change over time, and difference between generations.
- Here or in the methods a short description of the context of the cohort could be described, for example what are the common practices for school education for both generations in the majority of schools in the Avon area. Sexual education has shown to have great effects on some of the variables discussed and could have changed over time.

Methods:

- The main information I am missing in this description is whether the data of the generations can be linked. Can researchers see if the answers of the mother and/or the partner of the mother influence the answers of the children. Or is it only possible to look at the data as a type of related cross-sectional populations? The introduction seemed to suggest intergenerational comparisons, but the described is more broad than this.
- I have always been a bit baffled by the choice (also in other studies) to assess intercourse as a combination of vaginal, oral, and anal intercourse. Since their consequences are so different. For most attitudinal question I don’t think it matters much, but for example for the very first time (F5) it does matter.
- The enjoyment question was devised to have a positive ending to the section. I am wondering why enjoyment did not get the same treatment as regret. Why these two things were not mirrored. I think it would  have been very interesting (especially in sex positive times) to as about enjoyment in peoples very first time for example. And find out reason for enjoyment as well. I know after the fact there is not much to do about this though.
- Similarly, it would be interesting to see whether people used alcohol etc in their last time sex, in order to compare this to the first time. This question almost seems to be a ‘blaming question’, you were early because you were disinhibited by alcohol. Order generations are more influenced in sexual decision by alcohol, but I think that could be offset by different cultural norms as well, especially as younger generations seem to drink much less than older generations.

Observations:

- In understand the choice to use sex at birth, it would be interesting to present a little data on where this is different from gender.
- Table2a I am not sure why these demographics are described, why for example is number of brothers and sisters of G0 interesting? Education level might need a subscript for international readers (I am UK based but A level still means little to me), social class is unclear, is rurality based on postcode or reported.
- Table2b Total would be helpful, clarify if living with partner is for everyone or those with partner – link to relation status seems non-matching.
- Hat seems to be missing is for example religion of GO/G1, since this demographic directly links to some of the sexual questions.
- Where difference are described between sexes or generations or both or those tested, what tests are used for this, and maybe even report the results, to be able to assess effect sizes by the readers, some differences seem more relevant than others.
- For the correlation tables it would be more helpful to present the meaningful labels, versus the variable names, this way readers don’t have to search what these mean in the key, was not easy to read. You could have them number in the first column and then use the numbers for the first row, to keep it feasible.
- I would refer to the ‘risky behaviours’ as behaviours, I find this too negative, some of the things just describe what people did (where they met partners), other could be risky but might not be (early debut, drinking while debuting – both could involve condoms, so might not be risky at all).
- I appreciate this is a description of data, but some of the described behaviours could use a bit of nuance. Especially around the partner numbers. For example, ‘Six-times as many G1 males reported 10 or more partners (3.6%) vs 0.6% of G1 females.’ Is this and gender difference or could this be confounded with sexual orientation, where starkly different norms hold.
- Around regret, it is unclear whether people regret the actual sex act or whether they regret the consequences (unplanned pregnancy), I think these are 2 very different things, and might have some correlation, but could be independent.

Are sufficient details of methods and materials provided to allow replication by others?

No

Is the rationale for creating the dataset(s) clearly described?

Yes

Are the datasets clearly presented in a useable and accessible format?

Yes

Are the protocols appropriate and is the work technically sound?

Yes

Reviewer Expertise:

Health psychology, behaviour in infection prevention, focus on sexual health.

I confirm that I have read this submission and believe that I have an appropriate level of expertise to confirm that it is of an acceptable scientific standard, however I have significant reservations, as outlined above.

# Response

**Published:** 2026-03-26

**Authors:** Yasmin Iles-Caven

**Affiliations:** Bristol Medical School: Population Health Sciences, University of Bristol, Bristol, UK

Firstly, we thank you for your very helpful critique of the paper and hope this revised version is greatly improved.  In response to your specific comments:

**Background: Early debut or early activity could benefit from a definition here, what is early, or what is too early?**

 This is not really clear cut internationally and prone to difficulties.  In England, we suggest 16 years (being the legal age of consent for heterosexuals since 1885) as the cut point.  Early debut being those aged <16. We have clarified this in paragraph 1 of the Background.

**I struck me that the background was slightly negative. In recent years we see that the younger generations delay initiation of sexual intercourse (see for example https://doi.org/10.1080/00224499.2023.2297906).  I am not sure the introduction captures change over time, and difference between generations.**

 We agree this is a little negative. We have extended the Discussion (indicating older age at sexual debut becoming more prevalent; and that sexual experiences can have both positive and negative outcomes). Another paragraph therein briefly summarises why there may be a trend for older age at sexual debut.  Additional references are: de Graaf H, Schouten F, van Dorsselaer S, Költő A, Ball J, Stevens GW, de Looze M. Trends and the gender gap in the reporting of sexual initiation among 15-year-olds: A comparison of 33 European countries. The Journal of Sex Research. 2025; 62(4): 445-454. [Thank you] Golden, R. L., Furman, W., & Collibee, C. (2016). The risks and rewards of sexual debut. *Developmental Psychology*, 52(11), 1913. https://doi.org/10.1037/dev0000206 Hawes ZC, Wellings K, Stephenson J.  First heterosexual intercourse in the United Kingdom: a review of the literature. J Sex Res 2010; 47: 137-152. https://doi.org/10.1080/00224490903509399 Twenge JM, Park H. The decline in adult activities among US adolescents, 1976–2016. *Child Dev.* 2019; 90(2): 638–654. https://doi.org/10.1111/cdev.12930  

 Here or in the methods a short description of the context of the cohort could be described, for example what are the common practices for school education for both generations in the majority of schools in the Avon area. Sexual education has shown to have great effects on some of the variables discussed and could have changed over time.

 Three paragraphs have been added to the end of the Background around school sex education over time in Britain attempting to encompass the decades in which the parents were adolescents. Nothing could be found specifically relating to the county of Avon.  The first of these paragraphs gives the age of parents at delivery and their year of birth. Variables available for school sex education in Years 3 and 6 for the offspring cohort are now reported in Strengths and Limitations. Additional references: Simpson E. Sex Education: Looking to the Past to Inform the Present. History of Education Society 2020. Sex Education: Looking to the Past to Inform the Present - History of Education Society (accessed 06.01.2026) Board of Education Educational Pamphlet no. 119:  Sex education in schools and youth organisations. London: HMSO, 1943 (accessed 05.01.2026). https://education-uk.org/documents/boardofed/1943-sex-ed.html Pilcher J. School sex education: policy and practice in England 1870 to 2000. *Sex Educ. *2005; 5(2): 153–170. https://doi.org/10.1080/14681810500038848 Sex Education Forum - website

**Methods:** **The main information I am missing in this description is whether the data of the generations can be linked. Can researchers see if the answers of the mother and/or the partner of the mother influence the answers of the children. Or is it only possible to look at the data as a type of related cross-sectional populations? The introduction seemed to suggest intergenerational comparisons, but the described is more broad than this.**

 Yes – parent/child information can be linked. This is stated in the Abstract and Strengths and Limitations (inter-generational comparisons). A short sentence has been added to the second paragraph on ALSPAC within the Background section to clarify.

**I have always been a bit baffled by the choice (also in other studies) to assess intercourse as a combination of vaginal, oral, and anal intercourse. Since their consequences are so different. For most attitudinal question I don’t think it matters much, but for example for the very first time (F5) it does matter.**

 Whilst we agree with this point, ALSPAC had always specified ‘intercourse’ in this manner to align with other studies such as NATSAL. A sentence explaining this has been added to the paragraph immediately after the preamble to Section F under ‘Data Collection in 2022’.

**The enjoyment question was devised to have a positive ending to the section. I am wondering why enjoyment did not get the same treatment as regret. Why these two things were not mirrored. I think it would  have been very interesting (especially in sex positive times) to as about enjoyment in peoples very first time for example. And find out reason for enjoyment as well. I know after the fact there is not much to do about this though.**

 In retrospect this would have been ideal and indeed very interesting, but limitations of space and funding and having similar data to NATSAL and other studies for comparison resulted in not giving the same emphasis on enjoyment as with regret.

**Similarly, it would be interesting to see whether people used alcohol etc in their last time sex, in order to compare this to the first time. This question almost seems to be a ‘blaming question’, you were early because you were disinhibited by alcohol. Older generations are more influenced in sexual decision by alcohol, but I think that could be offset by different cultural norms as well, especially as younger generations seem to drink much less than older generations.**

 This is a good point. It is unclear when this shift in patterns of alcohol consumption between generations happened. The 2022 data will allow cross-generational comparisons with alcohol consumption in general, as well as alcohol/drug use prior to sexual debut (Table 7). Previously collected data on the parent (G0) and offspring (G1) generations had a different focus.  For the parent generation the focus was on potential sexual abuse/assault in childhood and then on the quality of their sexual relationship post-delivery and up to 19 years post-partum (see Iles-Caven & Golding, 2024a).  Much more detailed information was collected on the offspring from the age of 11 including various degrees of physical experiences within the previous year (hugging, kissing, touching etc, through to intercourse), with reasons why (in love etc), if they wanted it to happen and if drugs or alcohol had been consumed prior to the experience.  Note these experiences were asked as happening within the previous year and not specifically to sexual debut (Iles-Caven & Golding, 2024b).  For this reason, we collected the data described in this data note.  There are inconsistencies over time in the wording of questions from experiences ‘at sexual debut’ to ‘within the last 12 months’ to ‘the last time you had sex’. 

**Observations:** In understand the choice to use sex at birth, it would be interesting to present a little data on where this is different from gender.

 We use sex assigned at birth for the offspring in order to make direct comparisons with their parents (of which very few were in same sex relationships at enrolment/delivery).  Data on an individual’s reported sexual identity and attraction to the same/opposite sex are available within this paper (Tables 3 and 4), e.g. for those G1 male (assigned at birth) offspring, 94 (6.9%) stated they were homosexual. N.B. A typo in Table 3 has been corrected. In addition, we have added Table 10 which reports on new data collected in the winter of 2022 from the G0 participants only, which became available after this paper was initially published.  This reports on gender identity.

**Table2a I am not sure why these demographics are described, why for example is number of brothers and sisters of G0 interesting?  Education level might need a subscript for international readers (I am UK based but A level still means little to me), social class is unclear, is rurality based on postcode or reported.**  

 We give these fairly basic demographics to show the diversity of the cohort who responded to the 2022 data collection.  We felt noting siblings (older/younger - from which can be ascertained whether the respondent was the eldest), was important as these variables may influence age at sexual debut, sources of information about sex and exposure to siblings’ romantic liaisons – as well as potential confounders such as coming from a large family which may limit parental supervision.  

 Table 2a also indicates that those continuing to participate in ALSPAC are more likely to be White, married at enrolment, and of higher SES and female (as emphasised in Strengths & Limitations). Footnotes have been added to this table explaining social class and education level. Rurality is based on postcode. This has been clarified in the text describing Tables 2a and 2b.  The footnotes are as follows:

- Table 2a
*Public exams, usually in 5–10 subjects, normally undertaken at the end of Year 11 (age 16). Formerly called ‘O’ (Ordinary) Levels, the current equivalent are GCSEs.
**Social class was coded using the OPCS jobs scale (I Higher professional/Managerial, II Lower professional/managerial, III Non-manual - skilled; III – Manual – skilled;  IV Manual – partially skilled, V Manual – unskilled) and dichotomized here into non-manual and manual.
***Urban/rural and IMD are based on postcode of the mother’s residence.
- Table 2b.
*GCSEs are national public exams, usually in 5–10 subjects, normally undertaken at the end of Year 11 (age 16), and formerly called ‘O’ (Ordinary) Levels.  ‘A’ (Advanced levels) are usually taken at 18 years and high grades in A levels are required for university entrance

**Table2b Total would be helpful, clarify if living with partner is for everyone or those with partner – link to relation status seems non-matching**.

- the first question asked if they currently had a partner and if yes, whether they lived with them. This has been clarified within the table.
- Figures will not necessarily match as not all individuals answered both questions and those completing the YPJ questionnaire (at 29 years) may not have gone on to complete the YPK questionnaire (at 30).  These reported frequencies are of those answering the 2022 questionnaires for whom we have data at 29 years.  This has been added as a note after Table 2b title.

**Hat seems to be missing is for example religion of GO/G1, since this demographic directly links to some of the sexual questions.**

 The population of Avon at the time of enrolment into the study was predominantly Christian (Church of England).  Only about 7% were Roman Catholic and <1% Muslim. Data have been collected at intervals (including antenatally) on religious beliefs in the G0s, and from the G1 cohort more recently.  This is described in other data notes, listed below. These data were not included in the demographics tables which were not designed to be an exhaustive list of potential confounders.  Whilst many respondents stated they were CofE, further data shows that they were not necessarily practising or even believed in God or a divine being.  

- Iles-Caven Y, Bickerstaffe I, Northstone K and Golding J. Spiritual and religious beliefs and behaviour: data collected from 27/28-year-old offspring in the Avon Longitudinal Study of Parents and Children, 2019-2020. [version 2; peer review: 3 approved, 1 approved with reservations]. Wellcome Open Res 2024, 6:215 ( https://doi.org/10.12688/wellcomeopenres.17153.2)
- Iles-Caven Y, Gregory S and Matthews S. Coding definitions of participant religious, non-religious and spiritual beliefs in the Avon Longitudinal Study of Parents & Children (ALSPAC). [version 1; peer review: 2 approved]. Wellcome Open Res 2023, 8:528 ( https://doi.org/10.12688/wellcomeopenres.20209.1)
- Iles-Caven Y, Gregory S, Bickerstaffe I et al. Parental spiritual and religious beliefs and behaviour data collected from the Avon Longitudinal Study of Parents and Children, 2020 [version 3; peer review: 3 approved]. Wellcome Open Res 2022, 6:175 ( https://doi.org/10.12688/wellcomeopenres.17010.3)
- Iles-Caven Y, Gregory S, Northstone K and Golding J. Longitudinal data on parental religious behaviour and beliefs from the Avon Longitudinal Study of Parents and Children (ALSPAC) [version 2; peer review: 2 approved]. Wellcome Open Res 2019, 4:38 ( https://doi.org/10.12688/wellcomeopenres.15127.2)

**Where difference are described between sexes or generations or both or those tested, what tests are used for this, and maybe even report the results, to be able to assess effect sizes by the readers, some differences seem more relevant than others.**       

  As a Data Note this is a purely descriptive paper and the only statistical analyses are the correlation coefficients reported in Tables 6a-6e, using STATA 18.

**For the correlation tables it would be more helpful to present the meaningful labels, versus the variable names, this way readers don’t have to search what these mean in the key, was not easy to read. You could have them number in the first column and then use the numbers for the first row, to keep it feasible.**  

 We have altered Tables 6a-6e as you suggested to make them clearer and avoid referring back to the key, which I’ve suggested to the editors could be omitted.

**I would refer to the ‘risky behaviours’ as behaviours, I find this too negative, some of the things just describe what people did (where they met partners), other could be risky but might not be (early debut, drinking while debuting – both could involve condoms, so might not be risky at all).**

 We altered ‘risky’ behaviours in various ways depending on location/context within the paper to ‘sexual behaviours’, or ‘potentially risky sexual behaviours’ etc.

**I appreciate this is a description of data, but some of the described behaviours could use a bit of nuance. Especially around the partner numbers. For example, ‘Six-times as many G1 males reported 10 or more partners (3.6%) vs 0.6% of G1 females.’ Is this a gender difference or could this be confounded with sexual orientation, where starkly different norms hold.**

 Good point – However, we do not wish to speculate why this might be and leave it to interested researchers to analyse the data.

**Around regret, it is unclear whether people regret the actual sex act or whether they regret the consequences (unplanned pregnancy), I think these are 2 very different things, and might have some correlation, but could be independent.**

 We agree with you. It would be difficult to tease apart unless looking at the text responses – some are clearly related to sexual debut (“the first time it was painful”; “I wished I’d known more about what to do”) but many are clearly not or are ambiguous (“it was a violent relationship”). In retrospect, the layout of questions could have been better with a separate reason list for regret at debut vs. lifetime regret.  The following text has been added as a paragraph before Table 9b:

 Unfortunately, from the available data, it is difficult to ascertain whether respondents regretted the sexual act itself or the consequences of it.  Text responses (Table 9b) are not particularly helpful in this regard, some are clearly concerned with sexual debut (e.g. ‘the first time it was painful’), but others are more ambiguous (e.g. ‘it was a violent relationship’). In retrospect, the layout of the questions could have been much improved with separate tick box lists for regret at debut vs lifetime regret.
